# Phylogenomic analysis of natural selection pressure in *Streptococcus *genomes

**DOI:** 10.1186/1471-2148-7-154

**Published:** 2007-08-30

**Authors:** Maria Anisimova, Joseph Bielawski, Katherine Dunn, Ziheng Yang

**Affiliations:** 1 Department of Biology, University College London, London, UK; 2 Department of Biology, Dalhousie University, Halifax, Nova Scotia, Canada; 3 Department of Mathematics and Statistics, Dalhousie University, Halifax, Nova Scotia, Canada

## Abstract

**Background:**

In comparative analyses of bacterial pathogens, it has been common practice to discriminate between two types of genes: (i) those shared by pathogens and their non-pathogenic relatives (core genes), and (ii) those found exclusively in pathogens (pathogen-specific accessory genes). Rather than attempting to *a priori *delineate genes into sets more or less relevant to pathogenicity, we took a broad approach to the analysis of *Streptococcus *species by investigating the strength of natural selection in all clusters of homologous genes. The genus *Streptococcus *is comprised of a wide variety of both pathogenic and commensal lineages, and we relate our findings to the pre-existing knowledge of *Streptococcus *virulence factors.

**Results:**

Our analysis of 1730 gene clusters revealed 136 cases of positive Darwinian selection, which we suggest is most likely to result from an antagonistic interaction between the host and pathogen at the molecular level. A two-step validation procedure suggests that positive selection was robustly identified in our genomic survey. We found no evidence to support the notion that pathogen specific accessory genes are more likely to be subject to positive selection than core genes. Indeed, we even uncovered a few cases of essential gene evolution by positive selection. Among the gene clusters subject to positive selection, a large fraction (29%) can be connected to virulence. The most striking finding was that a considerable fraction of the positively selected genes are also known to have tissue specific patterns of expression during invasive disease. As current expression data is far from comprehensive, we suggest that this fraction was underestimated.

**Conclusion:**

Our findings suggest that pathogen specific genes, although a popular focus of research, do not provide a complete picture of the evolutionary dynamics of virulence. The results of this study, and others, support the notion that the products of both core and accessory genes participate in complex networks that comprise the molecular basis of virulence. Future work should seek to understand the evolutionary dynamics of both core and accessory genes as a function of the networks in which they participate.

## Background

The large number of complete bacterial genomes in the public domain (> 390) has opened the way for genome-scale studies of pathogenesis, diversity, evolution and gene function. There has been particular interest in comparative analyses of bacterial pathogens, and especially studying molecular mechanisms underlying bacterial adaptation, as such analyses are expected to contribute to disease prevention and treatment [e.g., [1,2]]. A typical approach is to discriminate between exclusive subsets of genes; e.g., genes shared among pathogens and their non-pathogenic relatives (core genes) and those genes showing presence-absence polymorphisms (accessory genes) [3]. Most studies of pathogenicity have restricted themselves to pathogen-specific subsets of accessory genes [e.g., [1]]. Only recently has the potential role of core genes in the evolution of pathogenicity been considered [e.g., [2]]. However, the outcome of dividing genes into exclusive subsets is sensitive to the particular sample of the genomes [4], and this could bias an investigation of the origin and maintenance of bacterial virulence.

This paper focuses on the evolution of genes and gene families of streptococci. The genus *Streptococcus *is comprised of a wide variety (about 48 species) of both pathogenic and commensal gram-positive bacteria, which are found to inhabit a wide range of hosts, including humans, horses, pigs and cows [5]. Within the host, streptococci often colonise the mucosal surfaces of the mouth, nares and pharynx, but also inhabit the skin, heart or muscle tissue. Niche or tissue-specific adaptations of streptococci remain poorly understood [6]. This study includes the three most important streptococcal human pathogens: *S. pyogenes *causes pharyngitis, wound and skin infections, scarlet and rheumatic fever, pneumonia, necrotising fasciitis, acute glomerulonephritis, cellulitis, and toxic shock syndrome [7]; *S. agalactiae *is known mainly for severe infections in newborns, such as sepsis, meningitis, and pneumonia, but also causes pneumonia and infections of bloodstream, skin, and urinary tract in adults [8]; and *S. pneumoniae *is a major source for pneumonia, meningitis, septicaemia, otitis media and sometimes occult bacteremia [9]. Most other streptococci are part of normal human flora [5], and three such lineages (two *S. thermophilus *and one *S. mutans*) are included in this study. Even within a nominal species, the genomes of individual strains vary in size and gene content (Table 1). Undoubtedly, some gene content variation among the lineages sampled in this study corresponds to important pathogenic differences between individual strains. Rather than attempting to *a priori *delineate subsets of streptococci genes into sets more or less relevant to pathogenicity, we adopted an approach focused on clusters of homologous genes. Although such clusters will not necessarily contain genes involved in presence-absence polymorphisms among pathogenic and non-pathogenic species, they will allow for a broader investigation of functional divergence among pathogenic and non-pathogenic lineages of streptococci.

**Table 1:** Twelve complete genomes of congeneric *Streptococcus *used in this study

***Streptococcus *strain**	**Genbank accession no.**	**Genome size (bp)**	**No. of CDs (>99 codons)**	**Reference**
*S. pyogenes *M1 GAS	NC_002737	1,852,441	1,697 (1509)	[60]
*S. pyogenes *MGAS8232	NC_003485	1,895,017	1,845 (1584)	[7]
*S. pyogenes *MGAS315	NC_004070	1,900,521	1,865 (1596)	[61]
*S. pyogenes *SSI-1	NC_004606	1,894,275	1,861 (1573)	[62]
*S. pyogenes *MGAS10394	NC_006086	1,899,877	1,886 (1582)	[63]
*S. agalactiae *NEM316	NC_004368	2,211,485	2,094 (1887)	[8]
*S. agalactiae *2603V/R	NC_004116	2,160,267	2,124 (1831)	[64]
*S. pneumoniae *R6	NC_003098	2,038,615	2,043 (1723)	[65]
*S. pneumoniae *TIGR4	NC_003028	2,160,837	2,094 (1701)	[66]
*S. mutans *UA159	NC_004350	2,030,921	1,960 (1690)	[67]
*S. thermophilus *LMG18311	NC_006448	1,796,846	1,889 (1541)	[68]
*S. thermophilus *CNRZ1066	NC_006449	1,796,226	1,915 (1554)	[68]

We estimated rates of evolution for gene clusters comprised of known and putative coding regions, categorised them according to their evolutionary patterns, and identified genes and gene families evolving under positive selection. We found that both core and accessory genes are subject to evolution by positive selection, most likely in the form of diversifying selection resulting from the antagonistic interaction between host and parasite. A large fraction of the genes identified as subject to positive selection have functions that can be connected to virulence. Furthermore, 19% of the positively selected streptococci gene clusters encode proteins that are known to have body-site specific expression pattern during invasive disease; a result which we argue is likely a substantial underestimate. Taken together, the results of this study and others support the notion that the products of both core and auxiliary genes participate in complex networks that comprise the molecular basis of virulence, and that evolution of the participants of such networks contribute significantly to the dynamics of this phenotype over time.

## Results and Discussion

### Characteristics of streptococci gene clusters

We partitioned the twelve complete genomes from five *Streptococcus *species (Table 1) into 1730 clusters of homologous gene sequences based on the requirement that a cluster must be comprised of more than three distinct sequences and meet a sequence similarity threshold of 50%. When the threshold was lowered to 30%, 1822 homologous gene groups were identified, with 78 of them obtained by a successive clustering procedure (see methods). Both sets of clusters were subjected to identical data analysis procedures. Despite the clustering differences, the results for both sets were consistent: the same conclusions were drawn from the estimates of evolutionary parameters, and the sets of genes detected to be under positive selection were very similar. This indicates a level of robustness of our results to the details of the clustering procedure. We therefore show results using the set of homologous gene clusters based on the 50% similarity threshold (unless stated otherwise). In this case, the size of clusters varied from 3 to 59 sequences, and almost a third of clusters (574) contained at least 12 sequences. Sequence length ranged from 300 (the lower bound on our selection criterion) to 4944 nucleotides, the average being 1027 nucleotides.

As expected, the sampled streptococci genomes were AT-rich, with nucleotide content varying among different codon positions, and among genes and genomes. The average AT content of the sampled genomes ranged from 60 to 64%, and the average for the gene clusters ranged from 49–74%. AT content at 2^nd ^and 3^rd ^codon positions (65% and 68% respectively) was more biased as compared to 1^st ^codon positions (49%). For certain genes AT content was extremely high at 3^rd ^codon positions while overall AT content was comparatively low: the majority of these cases were 30S and 50S ribosomal proteins (*rpsA*, *E*, *I*, *K*, *M *and *rplB*, *D*, *J*, *K*, *L*, *O*, *Q*) as well as uridine phosphorylase (*udp*), protein GRAB (known virulence factor), cell division protein (*ftsZ*), alcohol dehydrogenase I (*adhA*, *P*, *1*), cysteine synthase, O-acetylserine sulfhydrylase (*cysM*, *M1*, *K*), triose phosphate isomerase (*tpi*), cell-wall surface anchor family protein and a homologous cluster containing glucan binding protein (*pcsB*), secreted antigen (*gbpB/sagA*), and general stress protein GSP-781.

Transition/transversion (*κ*) ratios varied widely among the gene clusters: 95% of gene clusters had *κ *between 1 and 20, with the average ratio being 2. At the tails of the distribution we also found 2.8% of gene clusters with no transversions (forcing the estimate of *κ *to the upper boundary) and 2% of gene clusters with *κ *< 1. The latter cases included B-cell receptor associated protein, minor capsid protein, peptidoglycan hydrolase (*mur1*), ABC transporter membrane-spanning permease (*fatD*; iron transport), bacterocin transport accessory (*bta*), oxidoreductase (*mocA*), acetylglutamate kinase (*argB*), and pyrroline carboxylate reductase (*proC*).

We used the cluster-specific gene trees, as estimated by PHYML [10], as the basis to estimate the mean number of changes per codon per branch under model M0, as implemented in PAML [11]. In 95% of the clusters the mean amount of codon evolution was between 0.003 and 8 substitutions per codon site. However, in a small fraction of clusters (0.5%) we observed either a very low amount of codon evolution (<0.001 for orthologues from the same species, *S. pyogenes*), or a very high amount of codon evolution (>20 due to excess of synonymous substitutions in orthologues from different species).

Extreme outliers for AT content, transition/transversion ratio, and high amount of codon evolution are strong candidates for genes having a history of lateral gene transfer between members of the genus *Streptococcus *and more distantly related outgroup lineages [e.g., [12,13]]. We note however, that a large amount of codon evolution might also arise as a consequence of strong positive Darwinian selection.

### Phylogenomic analysis of evolutionary relationships among lineages

**Figure 1:**
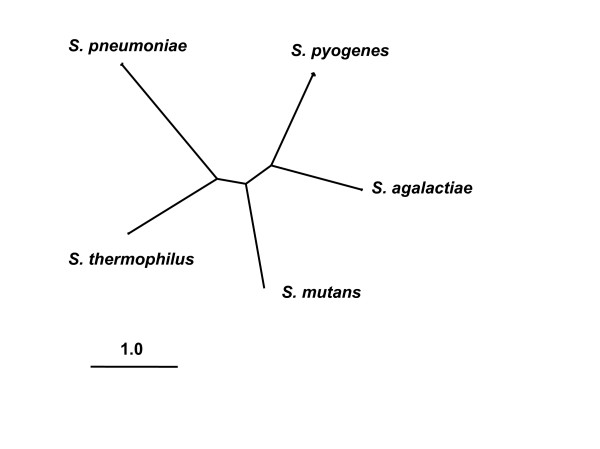
A genome-scale estimate of phylogenetic relationships among species of *Streptococcus*. The tree topology is derived from the joint analysis of 504 single-copy gene sequences by using the BIONJ algorithm [55]. Each gene was comprised of exactly one sequence from each of the 12 genomes; the topology shown above is simplified to show only the relationships among the five nominal species of *Streptococcus*. The topology is unrooted, and branch lengths indicate the mean number of substitutions per codon, as inferred under codon model M0 [11].

A subset of the gene clusters were concatenated for the purpose of obtaining a genome-scale estimate of the *Streptococcus *phylogeny. We restricted our phylogenetic analysis to the 504 clusters that were comprised of exactly one gene from each of the 12 genomes, as identified under the 30% threshold. Gene sequences from those 504 clusters were concatenated (555,297 nucleotides), and a phylogenetic analysis of this dataset resulted in the phylogenetic tree shown in Figure 1. This genome-scale estimate of relationships among species agrees with some prior estimates based on smaller molecular datasets [14,15]. The position of the root can be deduced by using the outgroup species *Lactococcus lactis*. Examination of the phylogenies estimated from each of the 504 single-gene clusters revealed that only 26% matched the species level relationship estimated from the concatenated data. Given such a high level of among gene variability, and the possibility that some portion could reflect inter-species recombination events [e.g., [16]], all subsequent analyses were based on cluster-specific estimates of the gene tree.

The length of the tree estimated from the concatenated data was 1.75 nucleotide substitutions per site. Tree length taken as an average over all the single-copy gene clusters was biased by the presence of trees with long lengths, where estimation problems were likely. After excluding 5% of trees having the largest estimated length, the average tree length was 3.53 nucleotide substitutions per site, with 95% between 1.4 and 51.2. According to simulations [17] high accuracy of ML estimation, and the LRTs, is expected under such levels of sequence divergence. The shortest trees (0.8 to 1.5) were obtained from 30S, 50S ribosomal proteins (*rpsB*, *C*, *D*, *G*, *K*, *L*, *M *and *rplC*, *E*, *N*, *P*, *Q*, *T*, *V*) and translation elongation factors (*tufA*, *fusA*). The longest trees (≥50) were obtained from membrane-spanning proteins, cell division proteins (*divlC*, *ftsL*), ATP-NAD kinase (*ppnK*), pantothenate kinase (coaA), diacylglycerol kinase (*dgk*, *dgkA*, *dagK*), exfoliative toxin A, exotoxin B (*eetB*, *shetA*), 3-dehydroquinate dehydratase (*aroD*), enoyl CoA hydratase II (*phaB*), and isopentenyl diphosphate isomerase (*idi*, *fni*). For genes of such high divergences the LRT has almost no power to detect positive selection, although the Bayesian prediction of sites under positive selection is expected to be highly unreliable in such cases [17,18].

### A two-step "genome scan" indicates robust identification of positive selection

Gene clusters identified as subject to positive selection pressure are listed in Additional file 1. We considered the signal of positive selection to be strong when both LRTs (M1a versus M2a and M7 versus M8) were significant at 5%; 38 gene clusters met this criterion (see Additional file 1). An additional 98 gene clusters were identified by the LRT of models M7 and M8 at the 5% significance level (see Additional file 1). For the latter cases, it is possible that model M2a was too conservative to detect positive selection. Indeed, simulation studies indicate that the LRT of M1a versus M2a is less powerful than the LRT of M7 versus M8 in such cases [17].

Any genome-scale study that surveys for positive selection is susceptible to false positives arising from uncertainty in parameter estimation [17], a history of recombination [19,20] or from procedural artefacts such as alignment errors. However, such surveys provide a powerful means to generate novel biological hypotheses for further experimental and statistical analysis [e.g., [2]]. To assess the potential impact of such errors on our investigation, we performed a substantially expanded analysis on a sample of genes we detected to be evolving under positive selection. To minimise the effect of uncertainty in parameter estimation, we compiled a larger set of gene sequences. To avoid potential for procedural artefacts, we analysed the data "by hand" rather than using our tools to automate the process; this included careful visual inspection and editing of the sequence alignments. Lastly, we employed the methods of Wilson and McVean [21] to evaluate the possibility of positive selection while simultaneously allowing for a history of recombination.

Clusters of genes initially identified as subject to positive selection were pre-screened for those comprised of a small number of sequences. Eight genes were then randomly chosen from this subset, conditional on the availability of additional homologous sequences in GenBank. We chose the ninth gene, *rplS*, because positive selection was unexpected, and the tenth gene, IS1191 transposase, because it is found only in *S. thermophilus*. All ten datasets were expanded by sampling homologous sequences from additional lineages of streptococci. We note that the distribution of strains among the streptococcal species was comparable. Genes subjected to expanded data analyses are listed in Table 2. Reanalysis under the standard set of models revealed that all ten datasets retained a signal of positive selection, six of which were significant at 1%, and two were significant at 5%, under both of the LRTs (Table 2). The remaining two datasets, *rplS *and a hypothetical protein-coding gene homologous to *sib38*, were significant only under the LRT comparing M7 and M8. We next employed the approach of Wilson and McVean [21] to evaluate the possibility that both selection and recombination might have impacted the evolution of these data. In each case the results of this approach, which does not suffer from high false positive rates stemming from a history of recombination, confirmed positive Darwinian selection (data not shown).

**Table 2:** Expanded analyses of ten genes detected to be under positive selection in a genome scan

					***P*-value of LRT**			
							
**Gene**	**Species**	**N_1_**	**N_2_**	**L_nt_**	**M1a vs M2a**	**M7 vs M8**	***p***_1_**in M8**	ω **in M8**
Cell envelope proteinase (prtS);	*S. pyogenes*	5	28	4947	0.0000	0.0000	0.010	15.0
Lactocepin Dipeptidase (pepD)	*S. pyogenes*	8	20	1416	0.0002	0.0000	0.016	8.0
	*S. agalactiae*							
	*S. mutans*							
Ftsk/SpoIII family protein^a^	*S. pyogenes*	4	10	756	0.0000	0.0000	0.049	∞
Hypothetical protein	*S. pyogenes*	4	10	1098	0.0000	0.0000	0.017	27.5
IS1191 transposase (truncated)	*S. thermophilus*	4	6	372	0.0020	0.0020	0.087	25.0
Hypothetical protein^b^	*S. pyogenes*	3	13	1092	0.0491	0.0004	0.033	12.4
Hypothetical protein^c^	*S. pyogenes*	4	7	402	0.0214	0.0214	0.026	31.2
grab	*S. pyogenes*	3	11	735	0.0035	0.0034	0.062	15.5
rplS^d^	all	11	30	345	0.1254	0.0080	0.010	6.7
Hypothetical protein^e^	*S. pyogenes*	4	13	1023	0.0679	0.0442	0.18	2.6
Homologue of mac/sib38								

Notes: N_1 _indicates the number of gene sequences in the original gene cluster. N_2 _indicates the number of sequences in the expanded dataset. L_nt _is the length of the gene sequence in nucleotides. *p*_1 _indicates the proportion of codon sites estimate to be subject to positive selection under model M8.

ω is the parameter in the codon model M8 for the nonsyonymous to synonymous rate ratio (*d*_N_/*d*_S_).

Additional remarks:

^a ^Best non-GAS blastp hit: gb|AAK79835.1|AE007695_8 (AE007695) FtsK-like DNA segregation ATPase, YDCQ *B. subtilis *orthologue [*Clostridium acetobutylicum*];

^b ^Fibronectin-binding protein (probable antigen);

^c ^Best non-GAS blastp hit: gb|AAB97959.1| (U96166) ATP-binding cassette lipoprotein [*S. cristatus*];

^d ^May play a role in the structure and function of the aminoacyl-tRNA binding site;

^e ^Novel immunoglobulin binding protein; best blastp hit: sp|P11215|ITAM_human cell surface glycoprotein MAC-1 α subunit precursor (CR-3 α chain) (CD11B) (leukocyte adhesion receptor MO1) (integrin α-M) (neutrophil adherence receptor).

The two cases where the LRT comparing models M1a and M2a failed to detect a signal of positive selection appears to reflect the greater restrictions on the parameter space of M1a and M2a as compared with M7 and M8. M1a and M2a explicitly model a class of purely neutral sites (ω = 1) although it might not exist in a given data set; in which case sites having ω = 1 could reflect an artefact of averaging over sites subject to moderate positive selection and other sites subject to purifying selection. Indeed, when we examined the parameter estimates under M8 for these two genes we observed (i) a very large fraction of sites (80%–82%) were subject to very strong purifying selection (ω ≈ 0.006) or moderate purifying selection (ω ranging from 0.11 to 0.36) and (ii) the absence of a purely neutral site class. These empirical results are consistent with the results of a simulation study [17] which showed that the LRT of M1 and M2 are often less powerful than the LRT of M7 and M8 in similar cases.

In general, by expanding these 10 datasets we increased the numbers of lineages and levels of sequence divergence (Table 2) to values where the LRTs are expected to have low type I error rates [17]. This result, taken together with robust detection of positive selection (Table 2), suggests that positive section was reliably identified from our ten samples of streptococci gene sequences. We note that large scale surveys for recombination using traditional detection software [e.g., [22]] are possible, but such programs likely suffer from an inverse effect, i.e. recombination may be falsely detected due to the presence of positive selection. Unfortunately, it is computationally prohibitive to carry out the approach of Wilson and McVean [21] for all genes in a genome. For the time being, we suggest the two-step process carried out in this study will provide a valuable means to gauge the robustness of selected cases obtained from such "genomic scans".

### A large fraction of genes subject to positive selection are connected to virulence

Since both orthologous and paralogous genes can be included in the same gene cluster, positive selection detected in a cluster can be a result of various processes: adaptation of a species to optimise the process of infection, escape host immune response, adaptation to a different environmental niches (e.g., milk in *S. thermophilus*, or dental flora in *S. mutans*), and functional diversification of members of multi-gene families. Indeed, the 136 clusters identified as having evolved under positive selection were connected to a wide variety of functions (see Additional file 1), with only 10% having no ascribable function. Interestingly, 7% of the clusters were comprised of genes known to be essential to *S. pneumoniae *(see Additional file 1), i.e. those where experimental disruption was shown to be lethal [23], indicating that even essential genes can be subject to rapid evolution by natural selection. A substantial fraction of the clusters, (29%) had either a known, or hypothesised, connection to virulence in streptococci (see Additional file 1). We considered a gene to have a connection to virulence if it was related to survival, spread, or persistence within its human host [24,25]. In the remainder of this section we focus on a select subset of the virulence genes subject to positive selection.

Three genes, *grab*, *endoS*, and *ideS*, are well known to play a critical role in the ability of pathogenic streptococci to evade detection by the host immune system. The *grab *gene encodes the G-related α_2 _macroglobulin-binding protein (GRAB) which functions to inhibit host-mediated proteolysis, a defence process that is often activated by the host during invasive infection [26].

GRAB mediates the binding of human α-2M (the dominant proteinase inhibitor of human plasma) to the surface of *S. pyogenes *thereby conferring protection against proteolytic degradation of important virulence determinants [26,27]. EndoS is an extracellular protein, encoded by the *endoS *gene, which hydrolyzes N-linked oligosaccharides on the heavy chains of immunoglobulin G (IgG), thereby impacting IgG function and preventing opsinising antibodies from triggering a protective immune response to *S pyogenes *[28]. The highly specific activity of this endoglycosidase increases pathogen survival in human blood containing opsonising antibodies [28-30]. Finally IdeS (or streptococcal Mac-1) is a secreted cysteine proteinase that cleaves the heavy chain of IgG and thereby also inhibits the susceptibility of *S. pyogenes *to phagocytosis [31]. The IdeS protein is specific to IgG antibodies and has been found to be expressed in clinically important serotypes. In each case above, the function of the protein is to modulate a critical host defence molecule. This creates a situation where an evolutionary arms-race between pathogen and host is likely, with the host subject to intense selection pressures to avoid such modulation of its defence systems by the pathogen. Our finding that each of these genes has evolved under positive selection pressure is consistent with such an antagonistic interaction between host and pathogen at the molecular level, and indicates that such evolutionary interactions are characteristic of several different systems used by pathogenic streptococci to escape recognition by the host. In each of the above systems the positive fitness consequences to the pathogen to winning such an arms race appears to be increased survival in human blood [28,31].

Escape from immune recognition is just one aspect of the ability of a pathogen to successfully invade and colonise its host. By conducting a genome-scale analysis of positive selection we identified sets of genes, when taken together with data derived from alternative methods of investigating virulence factors, provided insights into other potentially important molecular systems connected to streptococci virulence. For example, heat shock genes have been connected to bacterial virulence [32-34]. The products of heat shock genes are typically thought of as contributing to a protective cellular response to cope with the stress-induced damage of proteins. As such, many heat shock proteins are molecular chaperones or ATP-dependent proteases that play important roles in protein folding, repair, and degradation [34-36]. However, Charpentier and coworkers [37] also reported that ClpC proteases plays a major role in processes related to streptococci virulence, including adherence to human cells and production of known virulence factors such as pneumolysin, autolysin A, CbpA, and other choline binding proteins. Recently, Ibrahim et al. [34] suggested ClpC may play a major role in the virulence of *S. pneumoniae*. Consistent with a potentially important role in virulence, we found ATP-dependent proteases encoded by the *clpL*, *clpC *genes have evolved under positive selection pressure. We note that a signature-tagged mutagenesis screen of *S. pneumoniae *also identified ClpC as a virulence factor [38], and that ClpC has been shown to contribute to the ability of *S. pneumoniae *to grow in the lungs and blood [34]. Interestingly, we identified several other genes known to have a role in responding to stress conditions (*relA*, *gls24 *and *atpD/atpB*) as having evolved under positive selection.

Efficient genome replication is essential for growth and survival of an organism, and polymerase complexes often fail to complete this task [39,40]. For some pathogenic bacteria this is especially important, as successful replication is thought to contribute to proliferation and efficiency of the colonisation of hostile environments [e.g., [41,42]]. Interestingly, we detected positive selection in several single-copy protein-coding genes connected to replication, recombination and repair proteins (single-copy genes *recG*, *recN*, *recR*, *recM*), and in two ribosomal proteins (rpsB and rplS) and two DNA-dependent RNA polymerases (rpoC and rpoB). Replication forks often stall and may collapse to generate new DNA ends that provoke recombination and induce genomic rearrangements [40,43]. Complete replication of the genome depends therefore on repair activities to remove or bypass lesions in the DNA, modulation of RNA polymerase to reduce conflicts with transcription, and recombination systems to rescue forks that have stalled or collapsed. In these cases the nature of selection pressure is far from clear. Selection pressure on these genes could reflect constraints on efficient genome replication during colonisation and proliferation in the hostile environment of the host [e.g., [41,42]], or perhaps the ability to recombine during mixed colonisation by unrelated strains [e.g., [44]]. Neither is it known if these genes might be involved in an antagonistic interaction between host and pathogen at the molecular level.

Generally speaking, our genome-scale analysis of streptococci protein-coding genes uncovered a wide array of clusters that can be connected to virulence and which are subject to positive selection pressure. In some cases the nature of the selection pressure seems clear, e.g., *grab*, *endoS*, and *ideS *described above. We documented other cases where we identified genes previously hypothesised to play an important role in virulence but where current data were not conclusive. Yet another example of this is the two-component signal transduction systems mediated by histidine kinase; they are integral parts of bacterial cellular regulatory processes, and are used to regulate the expression of genes involved in virulence [25,45]. We identified five clusters comprised of genes from two-component response regulation systems, with two cases (*ihk and ciaR*) having been previously hypothesised to play an important role in virulence [34,46,47]. We believe that large-scale analyses such as ours play an important role in highlighting particular molecular systems that warrant further study. Finally, there were some cases where the nature of selection pressure was unclear, e.g., replication, recombination and repair proteins *recG*, *recN*, *recR*, *recM *described above. Identification of such genes helps to generate new hypotheses concerning the role of different molecular systems in virulence.

### Both the accessory genome and the core genome are subject to positive Darwinian selection

A typical approach to studying the evolution of pathogenicity is to focus on the subset of genes found exclusively in the genomes of pathogens [e.g., [1]]. Hereafter we refer to these genes as pathogen-specific accessory genes. Clearly the acquisition of such accessory genes by lateral gene transfer (LGT) plays an important role in the acquisition of characteristics necessary for a pathogenic lifestyle [e.g., [1,3]]. Since pathogens are typically involved in a co-evolutionary arms race with their hosts [48], one might expect pathogen-specific accessory genes are more likely to be subject to diversifying selection. Indeed, a recent study suggested that among recently transferred genes in *S. pyogenes *there is a higher relative frequency of positive selection [15]. To test if this notion is broadly applicable to all pathogen-specific genes we compared clusters of pathogen-specific accessory genes to clusters of core genes (clusters comprised of exactly one of each lineage of *Streptococcus*). Indeed, there were more pathogen-specific clusters (692) than core-gene clusters (526); however, the ratio of significant to non-significant cases for positive selection did not differ significantly between the pathogen-specific (44/648) and core gene clusters (41/485), as indicated by a Fisher's exact test (*P *= 0.364). We also tested pathogen-specific accessory genes against non-pathogen specific accessory genes, and found no evidence for a significant difference (*P *= 0.761). We note that a fraction of these gene clusters are likely have been impacted by gene duplication and deletion events, as well as by LGT.

Marri et al. [15] found that the relative frequency of positive selection was 3 fold higher in recently transferred genes in *S. pyogenes *as compared to core genes. As *S. pyogenes *causes a wider variety of human diseases than any other bacterial species, and possesses the largest number lineage-specific genes (381), we wanted to investigate the possibility that *S. pyogenes *also has a greater frequency of positive selection among its unique accessory genes. A chi-square test indicated that the odds of positive selection did not differ significantly among clusters of accessory genes unique to a given species of *Streptococcus *(*χ*^2 ^= 5.122, df = 4, *P *= 0.275). To minimise the impact of gene duplication and deletion, and to make a more direct comparison to the study of LGT by Marri et al. [15], we identified the clusters comprised of exactly one of each of the five sampled lineages of *S. pyogenes *and absent for all the other genomes under study. The relative frequency of positive selection was similar in both the single-copy *S. pyogenes *accessory genes (17/207) and in the streptococci core gene clusters (41/526), being about 8%. We note that Marri et al. [15] employed the same LRT (M7 against M8) to identify positively selected genes. It appears that the finding of higher odds of positive selection among recently transferred genes [15] could be sensitive to the method of assembly and classification of gene clusters.

As functional category has been shown to be related to selection pressure [49], we investigated the possibility that functional category could have been a confounding factor in our tests for heterogeneity between pathogen-specific gene clusters and core gene clusters. We subdivided clusters among 6 functional categories based on COG assignments and computed the odds of positive selection for pathogen-specific accessory gene clusters and core gene clusters (Table 3). Although the highest odds of positive selection among the pathogen-specific clusters was associated with cellular processes and signalling, the odds ratio for this functional category was 1.04, indicating that a high frequency of positive selection also occurred among the core gene clusters in this category. The highest odds ratio in favour of pathogen-specific accessory genes (2.92) did not differ significantly from one (Table 3). Indeed, the only category in which the odds of positive selection differed significantly between pathogen-specific accessory genes and core genes was for metabolism; and, in this case the odds of positive selection were higher for core gene clusters (Table 3).

**Table 3:** Odds of positive selection in gene clusters categorised according to COG-derived functional assignments

	**Odds of positive selection**			**Pathogen verses core clusters**	
		
	**All clusters**	**Pathogen-specific clusters**	**Core-gene clusters**	**Odds ratio**	***P*-value**
**Information storage & processing**	0.07	0.05	0.08	0.61	0.565
**Cellular processes & signalling**	0.10	0.10	0.10	1.04	1
**Metabolism**	0.08	0.04	0.12	0.35	0.02158
**Gene with inadequately characterised function**	0.05	0.07	0.02	2.92	0.2802
**Not in the COG database**	0.08	0.08	0.00	Infinite	1
**Genes with multiple COG functions**	0.07	0.06	0.09	0.68	1

Notes: The odds of positive selection were computed as the relative frequently of genes under positive selection divided by the relative frequency of genes not under positive selection. The odds ratio is simply the ratio of the odds of positive selection in two different categories of genes. The categories were based on functional assignments found in the COG database. The hypothesis that an odds ratio differed from one was tested by using the Fisher's exact test.

Our findings clearly indicate that the genes involved in presence-absence polymorphism among lineages of streptococci do not provide a complete picture of the nature of pathogen evolution within this genus, as core gene clusters have a similar propensity for positive selection pressure as pathogen-specific gene clusters. The properties of the analytical models used in this study provide some insight into this finding. LRTs based on the site-models we used are best suited to the discovery of genes having rapid amino acid change over long periods of time [e.g., [17]]. Among streptococci, this is most likely to arise in response to the antagonistic evolutionary arms-race between pathogen and host. As such an evolutionary interaction leads to diversifying selection pressure, where fitness advantages are conferred by possessing an allele that is merely different from others in the population; the 136 genes identified as subject to positive selection pressure are the best candidates for such a mode of evolution. Hence, our results reflect that *Streptococcus *pathogen evolution may be thought of having at least two components: (i) the acquisition by LGT of accessory genes that confer characteristics necessary for a pathogenic lifestyle, and (ii) the long term evolutionary arms race between host and pathogen that impacts the evolution of certain core and accessory genes. We note that other modes of adaptive evolution are plausible, such as a burst of adaptive evolution in an accessory gene following an LGT event or long term divergent selection pressure between pathogenic and non-pathogenic lineages of streptococci, but they are not detectible by using the codon models employed in this study.

### A large fraction of positively selected genes have tissue specific patterns of expression

In vivo gene expression studies of invasive disease have provided insights into the response of bacteria to the host during the course of infection by revealing that known virulence factors, along with other types of genes, display body-site specific expression patterns [6,50]. Such knowledge has lead to prediction that several streptococci genes of unknown function contribute to virulence, with these predictions being confirmed by experimentation [6]. However, extensive body-site specific contributions to pathogenesis make it difficult to determine if there is a core set of virulence-related genes required by all streptococci, or how extensive such a set of genes might be. Given such variability, we were interested to determine (i) what fraction of positively selected genes, if any, exhibited body-site specific patterns of gene expression during invasive disease, and (ii) if the intensity of selection might depend on their putative cellular role, as defined by level of gene expression.

**Figure 2:**
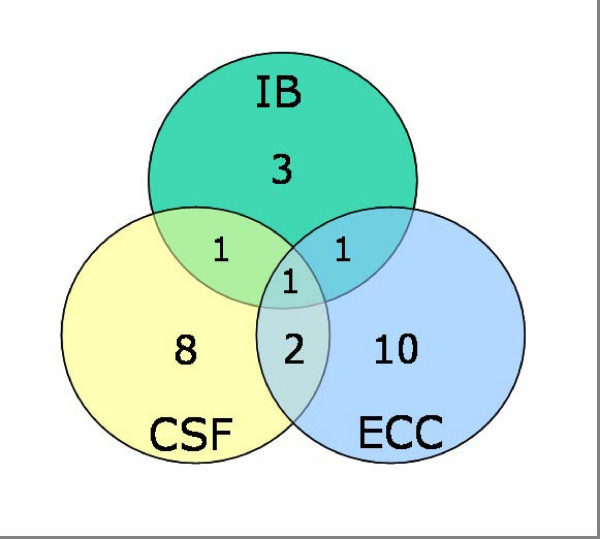
A Venn diagram showing the distribution of 26 proteins known to have body-site specific patterns of gene expression during invasive disease in *Streptococcus pyogenes *and to have evolved under positive Darwinian selection. Expression data are from Orihuela et al. [6] for infected blood (IB), cerebrospinal fluid (CSF) and epithelial cell contact (ECC).

We obtained expression data from the large-scale analysis of *S. pneumoniae *by Orihuela et al. [6] which provides data for expression levels within infected blood (IB), cerebrospinal fluid (CSF) and epithelial cell contact (ECC). Taken over all tissue types, 26 (19%) of the positively selected gene clusters encode proteins that are known to have body-site specific expression. However, the expression data is derived only from *S. pneumoniae*, and patterns of gene expression are thought to vary among pathogens. Interestingly, when we excluded the 47 gene clusters that did not contain at least one representative of *S. pneumoniae*, we found that the relative frequency of genes with body-site specific expression increased to 29%. The results are presented according to tissue-type in Figure 2. These data suggest a disparity among tissue types on the intensity of diversifying selection pressure, in so far as there were more positively selected genes having altered expression in CSF and ECC tissue types than in the other categories (Figure 2). Note that all the genes contributing to Figure 2 have >2 fold difference in tissue specific expression levels during invasive disease [6].

Current expression data is far from comprehensive, as it does not cover all body sites, variability among lineages of pathogens, nor even cover all the genes of *S. pneumoniae*. For this reason, we expect that we have underestimated the numbers of positively selected genes that have body-site specific expression during invasive disease. Large scale studies of expression level, signature tagged mutagenesis, and differential fluorescence induction are beginning to reveal that virulence is a function of a network of genes [e.g., [6,50,51]], many participants being core genes that are not well characterised. Indeed, our finding that core genes, and even essential genes, can be subject to an antagonistic evolutionary arms-race between pathogen and host also highlights the need to gain a better understanding of the role core gene products have in virulence. We look forward to the time when gene networks are better understood, particularly with respect to lineage-specific alterations in the pattern of transcription during invasive disease. An understanding of the evolutionary dynamics of virulence factors in such a context offers the potential to identify previously unappreciated targets for vaccines or pharmaceuticals [6,51].

## Conclusion

Based on our genome-wide analysis of selection pressure, we argue that the evolution of pathogen specific genes, although a popular focus of research, does not provide a complete picture of the evolutionary dynamics of virulence. There is no doubt that the acquisition of novel genes by LGT is critical to the colonisation and exploitation of novel niches, including the pathogenic lifestyle. However, those genes acquired by LGT impinge upon a complex network of interactions among gene products embedded in cellular metabolism. The effect of an LGT could be wide-spread in that the acquired gene or genes could have direct interactions with core gene products or result in an altered cellular environment (perhaps simply by allowing colonisation of a new niche) such that selective pressures acting on core gene products are impacted. As we gain a better understating of how niche specialisation, and pathogenicity in particular, is a function of networks of genes, we can begin to understand the evolutionary dynamics of individual core and auxiliary genes as a function of the networks in which they participate. The findings of this study, and others [e.g., [6,25]], suggest that the origin and evolution of the molecular mechanisms that result in enhanced virulence are more complex than previously thought.

## Methods

### Data, homologue clustering, and alignment

Twelve complete genomes of five *Streptococcus *species (available in Genbank) were analyzed in this paper: five strains of *S. pyogenes *(NC_004070, NC_003485, NC_002737, NC_006086, NC_004606), two strains of *S. agalactiae *(NC_004368, NC_004116), two strains of *S. pneumoniae *(NC_003098, NC_003028), one strain of *S. mutans *(NC_004350), and two strains of *S. thermophilus *(NC_006448, NC_006449). Table 1 summarises the key details of the sampled genomes. All known and putative codon regions longer than 100 codons were extracted from the genomes and the correct reading frame was preserved (i.e., every sequence started with the first codon position). None of the extracted sequences contained in-frame stop codons, and stop codons at the end of the sequences were deleted. The amino acid library was created by translating each DNA coding sequence into an amino acid sequence. We separated the amino acid sequences into homologous clusters by using BLASTCLUST program [52], which finds pairs of sequences that have statistically significant matches (using the BLAST algorithm) and groups them using single-linkage clustering. Two distinct sets of homologous clusters were produced based on minimum 30% and 50% similarity. Other clustering criteria were: 90% minimum length coverage and 10^-6 ^cut-off e-value. As a result, each cluster can contain both orthologous and paralogous sequences. Both sets of clusters were analysed using the procedure described below.

Each cluster of amino acid sequences was aligned using T-Coffee [53]. The alignments were based on BLOSUM62 matrix with gap opening and gap extension penalties -5 and -2 respectively. Codon alignments were created from the amino acid alignments by inserting three-nucleotide gaps into corresponding DNA sequences. Only clusters with at least three sequences were used for further analyses. Summary statistics were collected using PAUP*4.0 [54], and ML trees were reconstructed from DNA data using a heuristic search implemented in PHYML [10].

### Assessment of alignments

The set of clusters based on 30% similarity threshold were more divergent; consequently, for some large clusters alignments were problematic. In contrast, even the largest clusters based on the 50% similarity had acceptable alignments. In the absence of an objective universal score to compare alignments of different datasets, we chose the 15 largest alignments and visually inspected them for problems. Additionally, alignments for gene clusters from the highest divergence were visually inspected. All problematic alignments were discarded, and the involved data clusters were sub-clustered using BLASTCLUST with a higher similarity threshold. The similarity threshold was increased gradually with a step of 5% until a non-trivial sub-clustering was achieved. Sequences in new sub-clusters were re-aligned using procedures described above. These new alignments of sub-clusters were used in subsequent analyses.

### Species phylogeny

To investigate the phylogenetic relationships of the sampled strains of streptococci, we concatenated alignments from the 504 data sets which were created by using the 30% similarity threshold and which contained exactly one gene sequence from each of the 12 genomes. Such homologous clusters represent putative single-copy genes. The resultant 556,899 nucleotide-long alignment was used to reconstruct a NJ tree using fast BIONJ algorithm [55]. The branch lengths were estimated using the one-ratio codon model (M0) from PAML [11].

### Maximum likelihood (ML) analyses

The codeml program from the PAML [11] package was used for ML analyses based on Markov models of codon evolution. Branch lengths of the inferred ML trees, measured by number of expected nucleotide changes per codon, were estimated by using the simplest model M0 and then fixed in all further ML analyses to shorten the computational time. Model M0 assumes constant selective pressure across codon sites and over time. The selective pressure at the protein level was measured by *ω*, the ratio of nonsynonymous to synonymous rates *d*_N_/*d*_S_, with *ω *< 1, = 1, or > 1 indicating conserved, neutral or adaptive evolution respectively [e.g., [56]]. Models M1a, M2a, M7, and M8 of variable selective pressure across codon sites were used to estimate selective pressure and test for positive selection [57-59]. These models differ by the statistical distribution assumed for the *ω *ratio. For each homologous cluster, we performed two likelihood ratio tests (LRTs) for positive selection, comparing models that allow sites with *ω *> 1 (alternatives M2a and M8) with simpler models that do not (null models M1a and M7). Model M1a (nearly-neutral) assumes two site classes in proportions *p*_0 _and *p*_1 _= 1 - *p*_0_: one with *ω*_0 _ratio estimated between 0 and 1, and the other with *ω*_1 _fixed at 1. Alternative model M2a (positive selection) extends the null model M1a by adding a proportion *p*_2 _of positively selected sites with *ω*_2 _> 1, estimated from data. In the second LRT the alternative model M8 (beta&*ω*) extends the null model M7 (beta) by adding to the beta distribution for *ω *(defined on the interval [0, 1]), an extra class of sites under positive selection with *ω *> 1. To ensure convergence to the best likelihood, all analyses were performed three times. The analyses took 3–4 weeks on linux cluster comprised of 10 × 2.4 GHz AMD Opteron processors.

The minimum number of sequences for informative analysis depends on the divergence levels. Based on computer simulations [17], reasonable statistical power can be achieved with as few as 4 – 5 genomes for cases having divergences similar to those observed among *Streptococcus *strains.

Note that comparison of the absolute rate of synonymous (*d*_S_) or nonsynoymous (*d*_N_) substitutions among genes could be impacted by the way codon frequencies are handled [69]. However, it was not the purpose of this study to make quantitative comparisons among the genes; rather we sought simply to identify genes that had a history of positive diversifying selection. In this context, the interpretation of the *d*_N_/*d*_S _ratio should not have been affected [70]. In the case of LGT, extreme values for nucleotide content reflect differences in the equilibrium state of the donor and recipient genomes involved in the LGT event. The biggest factor for such genes will be a very large estimate of the amount of codon evolution among those lineages. As noted in Results and Discussion, a large amount of codon evolution is compatible with both the hypothesis of LGT and strong positive Darwinian selection.

## Authors' contributions

MA, JPB and ZY conceived the study. All authors participated in the design of the study. MA and KAD wrote the code that automated the genome-scale analyses, and carried out those analyses. All authors contributed to the statistical analysis and interpretation of results. MA, JPB and KAD were involved in drafting the manuscript. All authors have read and approved the final manuscript.

## Supplementary Material

Additional file 1 Gene clusters encoding proteins identified as having evolved under positive Darwinian selection pressure. Results of LRTs and additional information on gene clusters where positive selection was detected. Click here for file
