# Supplementary material for: Phylogenomic analysis of natural selection pressure in Streptococcus genomes
Source: BMC Evol Biol. 2007 Aug 30;7:154. doi: 10.1186/1471-2148-7-154 (PMC2031904; doi:10.1186/1471-2148-7-154)
Supplement: Additional file 1 — Gene clusters encoding proteins identified as having evolved under positive Darwinian selection pressure. Results of LRTs and additional information on gene clusters where positive selection was detected. [file 1471-2148-7-154-S1.doc]

**Gene clusters encoding proteins identified as having evolved under positive Darwinian selection pressure.**

|  |  |  |  |  | **LRT *P*-value** | |  |
| --- | --- | --- | --- | --- | --- | --- | --- |
| **Cluster ID** | **Protein** | **COG** | **#sequences (#pathogens)** | **t/br** | **M1a vs M2a** | **M7 vs M8** | **Notes** |
| 5 | Ribose-phosphate pyrophosphokinase | E F | 24(18) | 0.7462 |  | 0.0021 |  |
| 7 | Hyaluronidase - phage associated, Hyaluronoglucosaminidase | - | 20(20) | 0.1265 | 0.0045 | 0.0017 | a, b |
| 9 | Unknown phage protein | - | 19(19) | 0.2284 |  | 0.009 | a |
| 13 | General stress protein, Gls24 family similar to PID:2274940 | S | 18(16) | 4.5659 |  | 0.0023 |  |
| 16 | Formate-tetrahydrofolate ligase | F | 17(14) | 0.5782 |  | 0.0014 | b |
| 24 | Phage infection protein | D | 15(15) | 0.1183 | 0.0086 | 0.0005 | a |
| 31 | Carbamoyl-phosphate synthase, small subunit | E F | 14(11) | 0.8209 |  | 0.0002 |  |
| 34 | Positive transcriptional regulator | K | 14(12) | 0.3873 |  | 0.0355 |  |
| 37 | Aspartyl-tRNA synthetase | J | 13(9) | 0.4861 |  | 9.62E-06 |  |
| 38 | Possible ABC transporter, membrane component | O | 13(9) | 0.5976 |  | 0.0005 |  |
| 48 | Phosphoribosylformylglycinamidine synthase II (FGAM synthetase) | F | 12(9) | 0.1208 |  | 0.0275 | b, c |
| 49 | DNA-dependent RNA polymerase | K | 12(9) | 0.1255 |  | 4.33E-05 | b, c |
| 51 | DNA-dependent RNA polymerase subunit beta | K | 12(9) | 0.1429 |  | 0.0015 | b, c |
| 61 | P-type ATPase - calcium transporter; cation-transporting ATPase, E1-E2 family; Ca2+, Mn2+-P-type ATPase (homologous to yloB from Bacillus subtilis) | P | 12(9) | 0.3300 |  | 0.0086 | c |
| 73 | Exoribonuclease R, vacB/rnb family | K | 12(9) | 0.7508 |  | 0.0133 | b, c |
| 76 | Phenylalanyl-tRNA synthetase beta subunit | J R | 12(9) | 0.3628 | 0.045 | 0.0026 | c, d |
| 85 | Glycogen (maltodextrin) phosphorylase | G | 12(9) | 0.6007 |  | 0.008 | c |
| 88 | GTP pyrophosphokinase; (p)ppGpp synthetase | K T | 12(9) | 0.4748 |  | 0.0093 | c, d |
| 93 | Ribonucleotise-diphosphate reductase | F | 12(9) | 0.3504 |  | 0.019 | c |
| 96 | ATP-dependent protease ATP-binding subunit | O | 12(9) | 0.4687 |  | 0.00202 | b, c |
| 97 | Translation elongation factor G | J | 12(9) | 0.0870 |  | 0.0083 | c, d |
| 101 | ATP-dependent DNA helicase | K L | 12(9) | 0.3787 |  | 0.0027 | b, c |
| 113 | GTP-binding protein (tyrosine phosphorylated protein A); elongation factor Tu family protein | T | 7(7) | 0.1463 |  | 0.0483 | b |
| 121 | Group B oligopeptidase; Oligoendopeptidase F | E | 12(9) | 0.6066 |  | 0.0029 | c |
| 129 | DNA repair and genetic recombination protein | L | 12(9) | 0.6685 |  | 2.48E-05 | c |
| 156 | UDP-N-acetylglucosamine pyrophosphorylase | M | 12(9) | 0.4411 |  | 0.0055 | c, d |
| 164 | Phosphoribosylamine-glycine ligase | F | 12(9) | 0.1265 |  | 0.0061 | b, c |
| 166 | F_0_F_1_ membrane-bound proton-translocating ATPase, b-subunit | C | 12(9) | 0.1611 |  | 0.0119 | c |
| 169 | tRNA methyltranferase TrmA family | J | 12(9) | 0.7906 |  | 0.0083 | c |
| 177 | Glutathione reductase | C | 12(9) | 0.9032 |  | 0.0003 | c |
| 191 | Serine hydroxymethyltransferase | E | 12(9) | 0.5093 |  | 0.0426 | b, c |
| 213 | ABC transporter-associated protein, hypothetical | O | 12(9) | 0.4390 |  | 0.04218 | c |
| 221 | Serine/alanine adding enzyme | V | 12(9) | 1.3375 |  | 0.0459 | b, c |
| 223 | Aminotripeptidase; tripeptidase | E | 12(9) | 0.6465 |  | 0.001 | c |
| 227 | Oxygen-independent coproporphyrinogen III oxidase; Coproporphyrinogen oxidase, anaerobic | H | 12(9) | 0.3671 |  | 0.0376 | c |
| 233 | Phage-associated cell wall hydrolase; N-acetylmuramoyl-L-alanine amidase putative holin - phage associated; | N U | 12(12) | 0.1012 | 0.0124 | 8.46E-05 | a |
| 234 | Phosphoglycerate kinase | G | 12(9) | 0.0841 |  | 0.0018 | c, d |
| 270 | NAD(P)H-dependent glycerol-3-phosphate dehydrogenase | C | 12(9) | 0.7167 |  | 0.0115 | b, c |
| 290 | Branched chain amino acid aminotransferase | E H | 12(9) | 0.3472 |  | 0.0017 | c |
| 294 | Phospho-N-acetylmuramoyl-pentapeptide transferase; undecaprenyl-phosphate-UDP-MurNAc-penta peptide phospho-MurNAc-pentapeptide transferase | M | 12(9) | 0.7722 |  | 0.01842 | c, d |
| 298 | CMP-binding factor | R | 12(9) | 0.6302 |  | 0.0349 | c |
| 311 | Competence protein CglA; ABC transporter, ATP-binding protein ComYA; late competence protein, ABC transporter subunit | N U | 12(9) | 0.6013 |  | 0.03545 | c |
| 401 | 30S ribosomal protein S2 | J | 12(9) | 0.0916 |  | 0.0296 | b, c |
| 441 | ABC transporter membrane-spanning permease | P | 12(9) | 1.3328 | 0.0112 | 0.0376 | c |
| 443 | Putative 3-dehydroquinate dehydratase | E | 12(9) | 21.6883 |  | 0.0198 | c |
| 467 | Orotate phosphoribosyltransferase | F | 12(9) | 0.9229 |  | 0.0062 | b, c |
| 478 | Thymidine kinase | F | 12(9) | 0.4116 |  | 0.0313 | c |
| 502 | Transcriptional regulator, TetR/AcrR family | K | 12(9) | 3.8509 |  | 0.0328 | c |
| 514 | 16S rRNA processing protein | J | 12(9) | 0.7239 |  | 0.0278 | c, d |
| 531 | Transcriptional regulator, MarR family | K | 12(9) | 0.7986 |  | 0.0166 | c |
| 545 | Diacylglycerol kinase | M | 12(9) | 2.5463 |  | 0.0286 | c |
| 568 | Putative DNA-binding protein, signal recognition particle associated protein | S | 12(9) | 1.2994 |  | 0.0083 | c, d |
| 610 | Glycosyl transferase | M | 11(8) | 0.5514 |  | 0.0001 |  |
| 613 | ABC transporter sugar permease protein; carbohydrate ABC uptake transporter membrane-spanning protein; ABC transporter membrane-spanning permease - ribose/galactose transport | R | 11(8) | 0.6549 |  | 0.0293 |  |
| 617 | Fructose-1-phosphate kinase | G | 11(8) | 0.6748 |  | 0.0061 | b |
| 630 | The type 2 capsule locus of *Streptococcus* *pneumoniae*; glucose-1-phosphate thymidyl transferase | M | 11(8) | 0.2925 |  | 0.0476 |  |
| 648 | Hydroxyacylglutathione hydrolase/ conserved hypothetical protein, metallo-beta-lactamase superfamily | R | 11(8) | 0.4395 |  | 0.0192 |  |
| 685 | 50S ribosomal protein L19 | J | 11(8) | 0.1741 |  | 0.0097 | b |
| 686 | Hypothetical protein | S | 11(8) | 0.7695 |  | 0.043 | b |
| 695 | Formate acetyltransferase (pyruvate formate-lyase 2); formate acetyltransferase 3 | C | 10(9) | 0.5273 |  | 0.0005 | b |
| 698 | PTS system, glucose-specific IIABC component | G | 10(9) | 0.5197 |  | 0.0055 | b |
| 706 | Cationic amino acid transporter - APC Superfamily; amino acid permease family protein | E | 10(7) | 0.8138 |  | 0.0188 |  |
| 726 | Two component histidine kinase (homologue to HK03 Spn); | T | 12(9) | 1.0958 |  | 0.0126 | c |
| 736 | Hexulose-6-phosphate isomerase | G | 10(9) | 0.3512 |  | 0.002 |  |
| 737 | Hydrolase (HAD superfamily)/ hydrolase, haloacid dehalogenase-like family | R | 10(7) | 0.6563 |  | 0.011 |  |
| 743 | Methyltransferasel | R | 10(8) | 1.4112 |  | 0.0366 |  |
| 760 | DNA-binding response regulator, transcriptional regulatory protein | K T | 10(9) | 1.1335 |  | 0.0318 | b |
| 775 | Recombination protein | L | 10(7) | 2.6550 |  | 0.0378 |  |
| 781 | Peptide methionine sulfoxide reductase | O | 10(7) | 0.5401 |  | 0.00805 |  |
| 798 | Hypothetical (cytosolic) protein | S | 10(7) | 20.3166 |  | 0.0347 |  |
| 803 | Hypothetical (ribosome-associated) protein | J | 10(9) | 0.4702 |  | 0.0196 |  |
| 817 | 6-phospho-beta-galactosidase | G | 9(8) | 0.4254 |  | 0.0056 |  |
| 825 | Transcriptional regulator, GalR/LacI family | K | 9(9) | 1.3713 |  | 0.0175 | e |
| 827 | GMP reductase | F | 9(9) | 0.4675 |  | 0.0386 | e |
| 830 | ABC transporter membrane-spanning permease - sugar transport | G | 9(9) | 0.6643 |  | 0.029 | e |
| 874 | Polysaccharide biosynthesis protein; a-L-Rha a-1,2-L-rhamnosyltransferase | M | 8(5) | 1.8110 |  | 0.00608 |  |
| 875 | Putative ABC transporter, proline/glycine betaine permease protein | E | 8(7) | 0.3486 |  | 0.0341 | b |
| 878 | Putative amidase | J | 8(7) | 0.6757 |  | 0.0218 | b, e |
| 879 | Dipeptidase | E | 8(7) | 0.4259 | 0.0072 | 0.0044 | b |
| 893 | Iron ABC transporter, iron-binding protein | P | 8(8) | 0.9992 |  | 0.03086 |  |
| 901 | Integral membrane protein; hypothetical | S | 8(7) | 0.7556 |  | 0.0303 |  |
| 945 | Competence protein / late competence protein required for DNA uptake /DNA internalisation-related competence protein | R | 7(4) | 1.1792 | 0.0334 | 0.0053 |  |
| 954 | Multidrug resistance ABC transporter ATP-binding and permease protein; | V | 12(9) | 0.8698 |  | 0.008 | b, c |
| 974 | Homoserine dehydrogenase | E | 7(4) | 1.7314 |  | 0.0126 |  |
| 1040 | Putative two-component response regulator | T | 7(7) | 0.4091 | 0.0234 | 0.0135 |  |
| 1043 | Alpha-acetolactate decarboxylase | Q | 7(4) | 2.0284 |  | 0.0085 |  |
| 1047 | Branched chain amino acid ABC transporter ATP-binding protein | E | 7(4) | 0.6826 |  | 0.0469 |  |
| 1053 | Deoxyribose-phosphate aldolase | F | 7(7) | 0.0170 | 0.0007 | 0.0007 |  |
| 1084 | Transposase_A | L | 7(7) | 0.0608 | 7.01E-06 | 5.93E-06 | f |
| 1092 | Hypothetical protein | S | 6(5) | 0.6614 |  | 9.73E-07 |  |
| 1094 | Pneumococcal histidine triad protein D precursor; Histidine Motif-Containing protein | - | 6(6) | 0.1868 |  | 0.0158 | b, f |
| 1095 | Phage endopeptidase | E S | 6(6) | 0.0595 | 0.0363 | 0.0182 | a |
| 1100 | Putative citrate lyase, a-subunit/ Citrate CoA-transferase | C | 6(5) | 0.5043 |  | 0.043 | b |
| 1170 | Putative cell envelope proteinase Lactocepin similar to C5a-peptidase marked as predicted surface protein containing LPXTG-motif, | O | 5(5) | 0.0076 | 7.55E-06 | 7.40E-06 | a, b |
| 1175 | Secreted endoglycosidase; Endo-b-N-acetylglucosaminidase F2 precursor | G | 5(5) | 0.0862 | 1.33E-16 | 6.78E-17 | a, b |
| 1181 | Extracellular hyaluronate lyase; Hyaluronate lyase precursor | T U | 5(5) | 0.0261 | 1.13E-18 | 1.10E-18 | a, b |
| 1191 | Transcriptional antiterminator (BglG family); PTS system, mannitol (Cryptic)-specific IIA component | G K T | 5(5) | 0.0232 | 4.65E-13 | 3.96E-13 | a |
| 1192 | Putative platelet-binding protein - phage associated; Phage endopeptidase | S | 5(5) | 0.0572 | 0.0429 | 0.0407 | a, b |
| 1194 | Hypothetical (membrane spanning) protein | S | 5(5) | 0.0083 | 0.011 | 0.0109 | a |
| 1198 | ABC transporter, ATP-binding permease protein; lipid/multidrug/protein-type ABC exporter, ATP binding/membrane-spanning protein | V | 5(2) | 1.4926 |  | 0.0006 |  |
| 1199 | Hyaluronidase (phage associated); Phage infection protein | N Q R S | 5(5) | 0.0530 | 3.05E-14 | 2.68E-14 | a, b |
| 1222 | Minor capsid protein | L | 5(5) | 0.1787 | 1.70E-16 | 9.91E-17 | a |
| 1233 | Two component histidine kinase | T | 10(7) | 0.0067 |  | 0.0299 | b |
| 1260 | Hypothetical cytosolic protein | - | 5(5) | 0.0085 | 0.0256 | 0.0256 | a |
| 1295 | Hypothetical cytosolic protein | - | 5(5) | 0.0087 | 0.0316 | 0.0316 | a |
| 1302 | Anthranilate phosphoribosyltransferase | E | 5(2) | 2.5645 |  | 0.0277 |  |
| 1352 | Indole-3-glycerol phosphate synthase | E | 5(2) | 1.3154 |  | 0.0286 |  |
| 1358 | Hypothetical protein | - | 5(5) | 0.0144 | 3.88E-05 | 3.70E-05 | a |
| 1364 | Capsular polysaccharide biosynthesis protein Cps4B; exopolysaccharide biosynthesis protein | M G | 5(3) | 3.9819 |  | 8.21E-05 | d |
| 1376 | CAAX amino terminal protease family | R | 5(5) | 0.0099 | 0.0038 | 0.0037 | a |
| 1389 | Acetoin utilisation protein | R | 5(4) | 1.5096 |  | 0.0321 |  |
| 1400 | Formiminotetrahydrofolate cyclodeaminase/ putative serine cycle enzyme | E | 5(5) | 0.0631 |  | 0.0461 | a, b |
| 1422 | Hypothetical protein | S | 5(5) | 0.2940 |  | 0.014 |  |
| 1440 | Glutathione S-transferase | O | 5(5) | 0.0386 | 0.01862 | 0.0183 | a, b |
| 1446 | Histidine triad (HIT) protein, Bis-5'-nucleosyl-tetraphosphatase | F G R | 5(5) | 0.0267 | 0.0126 | 0.0123 | a |
| 1463 | Hypothetical protein | - | 5(5) | 0.0935 | 0.0101 | 0.01 | a |
| 1476 | Minor tail protein GP26; putative human platelet-binding protein, phage associated | S | 4(4) | 0.2387 | 0.0002 | 0.0001 | a, b |
| 1478 | Transcriptional antiterminator, BglG family sp2131 is marked as putative regulatory protein of pentitol metabolism | G K T | 4(4) | 0.6854 |  | 0.001 |  |
| 1483 | Terminase large subunit; hypothetical protein | R | 4(4) | 0.1653 |  | 0.0417 | a |
| 1496 | Two component histidine kinase | T | 4(4) | 0.0191 | 9.66E-08 | 8.44E-08 | a |
| 1516 | Late competence protein, ABC transporter subunit, | N U | 4(2) | 20.3511 |  | 1.21E-32 |  |
| 1517 | Hypothetical protein | - | 4(4) | 0.0180 | 1.60E-05 | 1.59E-05 | a |
| 1525 | IdeS gene | - | 4(4) | 0.1472 | 0.0012 | 0.0011 | a, b |
| 1552 | Putative oxidoreductase, short-chain dehydrogenase/reductase | R | 4(3) | 2.0538 |  | 0.0339 |  |
| 1557 | FtsK/SpoIIIE family protein | D | 4(4) | 0.0533 | 1.92E-11 | 1.89E-11 | a |
| 1561 | (Pyrogenic) Exotoxin G precursor | - | 4(4) | 0.0237 | 7.50E-07 | 8.95E-07 | a, b |
| 1606 | Hypothetical protein | - | 4(4) | 0.0373 | 0.0004 | 0.0003 | a |
| 1614 | IS1191, transposase, IS256 family, truncated | L | 4(0) | 0.0444 | 0.0004 | 0.0003 | g |
| 1647 | Na+/H+ antiporter | P | 3(1) | 5.0027 | 0.0097 | 0.0038 |  |
| 1648 | Putative transcriptional regulator, antiterminator | K | 3(2) | 2.3847 |  | 0.0432 |  |
| 1664 | Dextransucrase (sucrose 6-glucosyltransferase) | G | 3(2) | 1.2390 |  | 0.0111 |  |
| 1695 | Acetylornithine aminotransferase | E | 3(0) | 2.2005 |  | 0.0334 | g |
| 1710 | Hypothetical protein | - | 3(3) | 0.3873 | 0.0374 | 0.035 | a |
| 1715 | D-lactate dehydrogenase; putative D-specific D-2-hydroxyacid dehydrogenase | C H R | 3(3) | 2.2407 |  | 0.0499 |  |
| 1743 | Multiple sugar-binding ABC transporter, membrane-spanning permease | G | 3(2) | 16.6666 | 0.0031 | 0.0065 |  |
| 1775 | G-related alpha 2M-binding protein | - | 3(3) | 0.0318 | 0.0105 | 0.0075 | a, b |

Notes: a. Cluster containing *S*. *pyogenes* only. b. Gene connected to virulence. c. Cluster which contains one of each lineage. d. Essential gene [23]. e. Cluster that contains only pathogenic lineages. f. Cluster containing *S. pneumoniae* only. g. Cluster which contains only non-pathogenic lineages.
